# Antifungal activity of green synthesized selenium nanoparticles and their effect on physiological, biochemical, and antioxidant defense system of mango under mango malformation disease

**DOI:** 10.1371/journal.pone.0274679

**Published:** 2023-02-07

**Authors:** Muhammad Shahbaz, Abida Akram, Naveed Iqbal Raja, Tariq Mukhtar, Asma Mehak, Noor Fatima, Maryam Ajmal, Kishwar Ali, Nilofar Mustafa, Fozia Abasi

**Affiliations:** 1 Department of Botany, Faculty of Sciences, Pir Mehr Ali Shah Arid Agriculture University Rawalpindi, Rawalpindi, Pakistan; 2 Department of Plant Pathology, Pir Mehr Ali Shah Arid Agriculture University Rawalpindi, Rawalpindi, Pakistan; 3 Department of Botany, Lahore College for Women University, Lahore, Pakistan; 4 College of General Education, University of Doha for Science and Technology, Doha, Qatar; CSIR-National Botanical Research Institute, INDIA

## Abstract

Plant extract-based green synthesis of nanoparticles is an emerging class of nanotechnology that has revolutionized the entire field of biological sciences. Green synthesized nanoparticles are used as super-growth promoters and antifungal agents. In this study, selenium nanoparticles (SeNPs) were synthesized using *Melia azedarach* leaves extract as the main reducing and stabilizing agent and characterized by UV–visible spectroscopy, scanning electron microscopy (SEM), X-ray diffraction (XRD), energy-dispersive X-ray (EDX), and fourier transform infrared spectrometer (FTIR). The green synthesized SeNPs were exogenously applied on *Mangifera indica* infected with mango malformation disease. The SeNPs at a concentration of 30 *μ*g/mL were found to be the best concentration which enhanced the physiological (chlorophyll and membrane stability index), and biochemical (proline and soluble sugar) parameters. The antioxidant defense system was also explored, and it was reported that green synthesized SeNPs significantly reduced the biotic stress by enhancing enzymatic and non-enzymatic activities. *In vitro* antifungal activity of SeNPs reported that 300 *μ*g/mL concentration inhibited the *Fusarium mangiferae* the most. This study is considered the first biocompatible approach to evaluate the potential of green synthesized SeNPs to improve the health of mango malformation-infected plants and effective management strategy to inhibit the growth of *F*. *mangifera*.

## 1. Introduction

Mango (*Mangifera indica* L.) belongs to the family Anacardiaceae, is a popular and highly nutritive fruit crop [1]. Its pulp is a rich source of vitamins A and C along with sugars, proteins, organic acids, carbohydrates, ascorbic acid, and minerals [2]. Pakistan ranks as the 6^th^ largest mango-producing country in the world. The mango industry is the second main fruit industry of Pakistan, with an annual production of 1.72 million tons on an area of about 171.7 thousand hectares. Pakistan exports about 85,000 tons of mango valued at around $36.66 million per year [3]. Quick decline of mango is the most recent severe threat to the Pakistani mango industry due to various biological diseases [4–6]. Among the diseases, mango malformation is the most destructive disease in Pakistan caused by *Fusarium mangifera*. This pathogen has been destroying fruit yield and ultimately decreasing the country’s economy [7, 8]. Mango malformation is one of the severe problems that can occur in both vegetative and floral tissues and can become virulent, resulting in the loss of the entire crop [9]. Typical symptoms are length of primary axis and secondary branches of panicle, which makes flowers, appear in clusters. The flower buds are transformed into vegetative buds, and many small leaves and stems, characterized by appreciably reduced internodes, give appearance of a witches’ broom [10].

Different methods, such as the use of fungicides are currently being used to protecting the plants from *F*. *mangifera* [11]. The inappropriate use of fungicides harms the environment, and its long-term use has resulted in a large proliferation in drug-resistant pathogens that are far more pathogenic than wild strain pathogens [12]. Scientists are looking for alternate disease control methods which can act efficiently against these pathogens [13]. In this aspect, nanotechnology plays a promising role in controlling plant diseases [14]. Nanotechnology is a 21st-century discipline and has become a valuable novel approach to manage several environmental challenges through providing innovative and effective solutions [15–17]. Nanoparticles are used in a wide range due to their vast and differing properties including medical, pharmaceutical, industrial, and other fields [18, 19]. Metalloids, nonmetals, metallic oxides, and carbon nanomaterials are examples of nanomaterials that have shown activity in controlling plant diseases [20]. In fact, selenium is a practical and essential element for the body that has proved to contain strong antioxidant and anti-cancer Properties [21–23].). Selenium nanoparticles (SeNPs) with higher bioavailability, lower toxicity, and good absorption capacity than inorganic and organic forms have a wide application in medical diagnostics and nanobiotechnology [24, 25]. The reduction of oxidative stress, anticancer drug delivery carrier, protection against metal intoxication, and immunostimulatory effect are some important applications of SeNPs. Also, SeNPs were used as antioxidant agent, chemo-preventive agent, antimicrobial agent, antifungal agent, therapeutic agent, and photocatalysts [26–29].

Various methods can be used for nanoparticles synthesis. Currently, the preparation of NPs is mostly based on chemicals, which have been noted as fast but toxic, expensive and non-environment friendly [30]. Green synthesis by using plant extracts, on the other hand, has grown in popularity since it is eco-friendly, pure, inexpensive, and biocompatible [31]. Plant-mediated nanoparticle manufacturing is faster than that of other biological species since there is no need to maintain media and culture conditions [32]. Plant extracts may act as reducing agents in nanoparticle production as metal nanoparticles can be prepared by reducing metal ions [33]. Stress generates the production of reactive oxygen species (ROS), which affects plant differentiation, development, and metabolism by interacting with biological components [34]. Plant cells counteract the harmful effects of ROS using SOD, POD, flavonoids and phenolic [35–38].

Few investigations on the exogenous application of NPs in some plant species have been published thus far. The role of green synthesized SeNPs as antifungal potential, biochemical profiling, and various quality and productivity parameters in mango under the stress of malformation disease is still to be explored. This study has been designed to study the effects of green synthesized SeNPs on physiological, biochemical, and antioxidant parameters of mango under the stress of mango malformation disease. This study also focused on the antifungal activity of green synthesized SeNPs against the causal agent of mango malformation i.e. *F*. *mangifera*.

## 2. Materials and methods

### 2.1. Sample collection and extract preparation

Fresh and green leaves of *Melia azedarach* were taken from Pir Mehr Ali Shah-Arid Agriculture University Rawalpindi. Leaves were washed with tap water to remove impurities (dust and unwanted particles) and dried for a week at room temperature. The leaf extract was prepared according to the methodology of Fardsadegh et al. [39]. Fine powder of leaves 4.69 g was added into 100 ml distilled water and allowed to boil for 5 minutes on hotplate (Sr. No G150). After this, filtration was done by using filter paper (no. 1, Whatman, Clifton, NJ) three times to obtain the pure extract. Sterile conditions were maintained in each step of the experiment to get free contaminated and accurate results.

### 2.2. Green synthesis of selenium nanoparticles

The selenium nanoparticles (SeNPs) were green synthesized by following the methodology of Satgurunathan et al. [40]. The commonly used salt for SeNPs preparation was sodium selenite (Na_2_SeO_3_). The solution of Na_2_SeO_3_ (10 mM) was prepared by mixing the Na_2_SeO_3_ (1.25 g) in 500 mL of water (distilled) and heating at 80°C along with magnetic stirring at a hot plate (Sr # G150) for 30 min. The *Melia azedarach* leaves extract was used to reduce sodium salt to SeNPs. *M*. *azedarach* extract was prepared by boiling the leaves (4.69 g) in 100 mL of distilled water for 5 minute by following the protocol of Fardsadegh et al. [39]. The *M*. *azedarach* extract was steadily added in Na_2_SeO_3_ solution with constant boiling at 100°C until the brick red color appeared. The solution was then centrifuged at 14,000 rpm for 15 minute at 25°C. The pellet was collected, and this process was repeated thrice. The pellet was dried utilizing a Speed Vac concentrator. The resulting SeNPs were subjected to their characterization and then used in the form of a foliar spray on mango trees against the mango malformation disease.

### 2.3. Characterization of green synthesized SeNPs

The formation of green synthesized SeNPs from Na_2_SeO_3_ was examined by recording the UV-Visible spectrum at the National Center of Physics (NCP), Islamabad. The structural analysis of the green synthesized SeNPs was done by scanning electron microscopy (SEM) using a SIGMA model operated at 5 kV, magnification×10K, from the Institute of Space and Technology (IST), Islamabad. The elemental analysis of the green synthesized SeNPs was also performed at IST, Islamabad by using an energy-dispersive X-ray (EDX) detector (SIGMA model). The crystalline nature of the green synthesized SeNPs was determined using X-ray diffraction (XRD) at the NCP, Islamabad. FT-IR was used to investigate functional groupings (Perkin Elmer Spectrum 100 FT-IR Spectrometer). The IR (Infra-Red) spectra were recorded at a resolution of 4.0 cm^–1^ in the middle wavelength range of 4000–400 cm^–1^.

### 2.4. Evaluation of different concentrations of green synthesized SeNPs against mango malformation

The experiment was performed at “Gillani Mango Orchard” village of Ghari Kandi, District Bahawalpur (longitude: 71.286928, latitude: 29.293757), Punjab, Pakistan, during the summer of 2021. A total of 36 mango trees of two varieties (V1: Chanusa and V2: Langra, 18 tress each) affected by mango malformation disease were selected and were sprayed with concentrations (10, 20, 30, 40, and 50 *μ*g/mL) of green synthesized SeNPs (Table 1). The age of all mango trees of both varieties was 32 years and average canopy size was 28 feet. All trees were growing in a row with 24 m long. The SeNPs were applied as foliar spray by using an atomizer pressurized uniformly on all branches of trees showing symptoms and then tagged with red colored tape. After two months, the leaves samples were collected from all sides, from top to down position uniformly from all selected trees. Leaves were cut with scissors and packed in sample collection bags. They were put into sample collection basket contains bags of ice and stored in refrigerator at 4°C until the physiological and biochemical analysis.

**Table 1:** Experimental layout.

Variety	Treatments	Concentration	No. of Trees
V1 (Chaunsa)	T_1_	0 ppm (Control)	3
	T_2_	10 ppm + Disease	3
	T_3_	20 ppm + Disease	3
	T_4_	30 ppm + Disease	3
	T_5_	40 ppm + Disease	3
	T_6_	50 ppm + Disease	3
V2 (Langra)	T_1_	0 ppm (Control)	3
	T_2_	10 ppm + Disease	3
	T_3_	20 ppm + Disease	3
	T_4_	30 ppm + Disease	3
	T_5_	40 ppm + Disease	3
	T_6_	50 ppm + Disease	3
Total			**36**

#### 2.4.1 Assessment of physiological parameters

*2*.*4*.*1*.*1 Chlorophyll contents*. The chlorophyll contents of the leaves were measured by following the protocol of Bruinsma et al. [41]. Plant leaves (2 g) were ground in acetone (10 mL) and after complete grinding, filtration was done with the help of filter paper (no. 1, Whatman, Clifton, NJ). Absorbance was observed at 645, 652, and 663 nm wavelengths using a spectrophotometer (Model U–2900 Sr. No 26E82–018). The chlorophyll content was calculated using the following formula:

$$Total\;Chlorophyll\;Contents=(A_{652}\times 1000/34.5)$$ (1)

*2*.*4*.*1*.*2 Membrane stability index*. The membrane stability index (MSI) was measured by following the protocol of Sairam et al. [42]. The 100 mg discs of leaves taken from each treatment were inserted into test tubes and test tubes were placed in a water bath for 30 min at 40°C. The electrical conductivity (C1) was then measured using an EC meter. The electrical conductivity of the test tubes (C2) was measured after 10 min in a water bath at 100°C. The membrane stability index was calculated using the formula:

$$MSI=1-\frac{C1}{C2}\times 100$$ (2)

#### 2.4.2 Assessment of biochemical and antioxidant parameters

*2*.*4*.*2*.*1 Proline contents*. Proline content was determined by using a spectrophotometer by following the protocol of Bates et al. [43]. Leaves (0.2 g) were crushed with 3% sulfosalicylic acid (4 mL). Plant extract (2 mL) were added to separate test tubes and allowed to react with the ninhydrin and then frozen acetic acid (2 mL) was added. It was boiled in a water bath and after the formation of yellow color, toluene (4 mL) was added and mixed thoroughly until the upper colorful layer was formed. This layer was separated from the rest of the liquid in another set of test tubes, and absorbance was measured at 520 nm. The proline content was calculated by using Eq 3:

$$TPC(mg/g)=\frac{Sample\;absorbance\times Dilution\;factor\times k\;value}{Fresh\;weight\;of\;plant\;tissue}$$ (3)

*2*.*4*.*2*.*2 Soluble sugar*. Soluble sugar was estimated by following the protocol of Qayyum et al. [44]. Leaves (0.5g) were mixed with 80% ethanol (10 mL) and boiled for one hour at 80°C in a water bath. One mL of phenol (18%) was combined with extract (0.5 mL) in the test tubes and placed at room temperature. After mixing and shaking, the absorbance of each duplicate was checked at 490 nm wavelength using a spectrophotometer.


$$Soluble\;sugar=\frac{Sample\;absorbance\times Dilution\;factor\times k\;value}{Fresh\;weight\;of\;plant\;tissue}$$ (4)

*2*.*4*.*2*.*3 SOD activity*. The reaction mixture was composed of 130 mM methionine, 1 mM EDTA, 0.75 mM NBT, 0.02 mM riboflavin, and 50 mM phosphate buffer (pH 7). A blank was made in the same method, but instead of enzyme extract, phosphate buffer was used. The absorbance was measured at 560 nm with a spectrophotometer after exposing the blank and reaction mixture to fluorescent light for 7 min [36]. After then, the Lambert–Beer law was used to compute the SOD activity.


A=εLC (5)

Where *A* is the absorbance, *ε* is the extinction coefficient, *L* the length of wall, and *C* the concentration of enzymes.

*2*.*4*.*2*.*4 Peroxidase activity (POD)*. By following the protocol of Lagrimini [45]. POD activity was calculated. The reaction mixture (2 mL) contained 200 *μ*l H_2_O_2_ (27.5 mM), 200 *μ*l enzyme extract, and 1 mL of water. The absorbance was measured at 470 nm with a spectrophotometer after exposing the blank and reaction mixture to fluorescent light for 7 min.

*2*.*4*.*2*.*5 Total phenolic contents*. The plant extract (100 mL) was combined with folin–Ciocalteu reagent (0.75 mL), and incubated at 22°C for 5 min. The mixture was then given 0.75 mL of Na_2_CO_3_ solution and maintained at 22°C for 90 min. The sample’s absorbance was measured at 725 nm by spectrophotometer [36].

*2*.*4*.*2*.*6 Total flavonoid components determination*. 10 mg of quercetin was mixed in 80% C_2_H_5_OH and diluted several times. The resultant standard solution was combined with 0.1 mL of CH_3_CO_2_K (1M), 1.5 mL of ethanol (95%), 0.1 mL of AlCl₃ (10%), and 2.8 mL of distilled water at room temperature for 30 min. Then, by using spectrophotometer, the absorbance of the combination was measured at 415 nm [36].

### 2.5. *In vitro* assessment of antifungal activity and growth inhibition of green synthesized SeNPs

#### 2.5.1 Isolation and purification

The infected inflorescence of mango trees with mango malformation disease was subjected to fungus isolation by following the methodology of Caicedo et al. [46]. with some modification. The infected inflorescence was surface sterilized with sodium hypochlorite (2%) and ethanol (70%) followed by washing with sterile distilled water three times. The Potato Dextrose Agar (PDA, Oxoid, Basingstoke, UK) was made by dissolving 39g of PDA in 1 L of double distilled water and dissolved by heating and autoclaved at 121°C and 15 psi for 20 min, the medium was cooled at approximately 50°C and poured into 9 cm diameter Petri dishes [47, 48]. Infected parts were cut into small pieces, plated in petri plate and incubated for 3–5 days at 28±2°C for the emergence of colonies with an alternative light and darkness cycle in a growth chamber. After 3–5 days, fungal colonies arising from the plant were investigated under a light microscope (Olympus Optical CO., Ltd. Japan). The growing fungus was then transferred to new PDA plates. Single-spore or hyphal-tip purification procedures were used to purify the recovered fungus. Pure cultures of the recovered fungus were grown on PDA slants at 30 degrees Celsius before being stored as stock cultures in a refrigerator at 5 degrees Celsius. The identification of the fungus was made based on the growth pattern, conidia, and colony characteristics according to published procedures [49, 50].

### 2.6. *In vitro* antifungal activity of green synthesized SeNPs

#### 2.6.1 Well diffusion assay

The well diffusion assay was performed to study the antifungal activity of green synthesized SeNPs by following the methodology of Menon et al. [51]. with little modifications. The spore suspension of the fungus *F*. *mangifera* (1.5 x 10^8^ CFU/ mL) was thoroughly dispersed on the sterilized solidified PDA medium. Six wells of 5.5 mm diameter were made on each agar plate with the help of a sterile cork-borer. The wells were filled with 10 μl of different concentrations (150, 200, 250, 300 *μ*g/mL) of SeNPs individually with triplicates [52]. The plates were incubated at 25°C for seven days, and the zones of inhibition were measured. Antifungal drug Barresten tablets 500 mg were purchased from a local pharmacy and 200 *μ*g/mL of its concentration was put into well as a positive control. The Petri plates were placed in an incubator for one day at 37°C and 5 days at 28°C.

#### 2.6.2 Radial growth method

PDA medium was prepared and amended with different concentrations of SeNPs (150, 200, 250, 300 *μ*g/mL) before the pouring stage. For positive control, no concentration of SeNPs was added. Culturing of *F*. *mangifera* was carried out after medium solidification, according to Joshi et al. [53]. The inhibition percentage of pathogen growth was calculated using the following equation:

$$Inhibition\;of\;pathogen\;growth\;(\%)=\frac{Growth\;in\;the\;control-Growth\;in\;the\;treatment}{Growth\;in\;the\;control}\times 100$$ (6)

#### 2.6.3 *In vitro* leaflet assay

One-month-old mango leaves were surface sterilized with NaOCl (2%) and moistened with 300 *μ*g/mL SeNPs. When leaves got dried, 5mm disc of agar plug containing *F*. *mangifera* (causing agent of mango malformation in *Mangifera indica)* was placed in the center of each leaf. A leaf without priming with SeNPs was used as a positive control. The leaves were placed for 7 days at 22°C under dark conditions and the expansion of fungus was reported [53].

#### 2.6.4 Cavity slide test method

By following the protocol of Singh and Vyas [54], cavity slide test method was performed to check the influence of antifungal substances against the germination of spores of the pathogen (*F*. *mangiferae)*. For this, serial dilution was made and a density of spores at 10^6^ spores/ mL was used. The SeNPs concentration of 300 *μ*g/mL was used. SeNPs along with fungal spore suspension usually 100 *μ*l were injected into each cavity slide and cavity slides were subjected into a moist chamber at 28–30°C. After 24 hours of the incubation period, using a light microscope percentage value of spore germination was determined.

### 2.7. Statistical analysis

All the measured data were statistically analyzed using Statistics 8.1. To test the overall significance of the data ANOVA (Analysis of variance) was computed using the Least Significant difference (LSD) at 5% probability level for comparing mean values.

## 3. Results and discussions

### 3.1. Synthesis and characterization of SeNPs

The green synthesis of SeNPs was carried out using *Melia azedarach* leaves as the main reducing and stabilizing agent. Sodium selenite (Na_2_SeO_3_) stock solution was mixed with *M*. *azedarach* leaves extract. At the beginning, the reaction mixture was light green in color, later the color began to change, and finally brick red color was form which confirmed the formation of SeNPs. Certain concentration of active components is thought to be present in plant extract that is important in NPs synthesis [55]. The ability to synthesize SeNPs from plant extracts is important since it reduces downstream processing and the difficulty of maintaining cell cultures [56]. During the reduction reaction, the color of Na_2_SeO_3_ changed from colorless to brick-red, indicative of nanoparticles production [57]. The color of Na_2_SeO_3_ solution changed from colorless to brick red in our study, showing that SeNPs were biosynthesized from Na_2_SeO_3_ by *M*. *azedarach* leaves extract’s reduction and stabilizing activities. Our observations were like Satgurunathan et al. [40], who used Na_2_SeO_3_ and *Allium sativum* clove extract for the formation of SeNPs.

**Fig 1:**
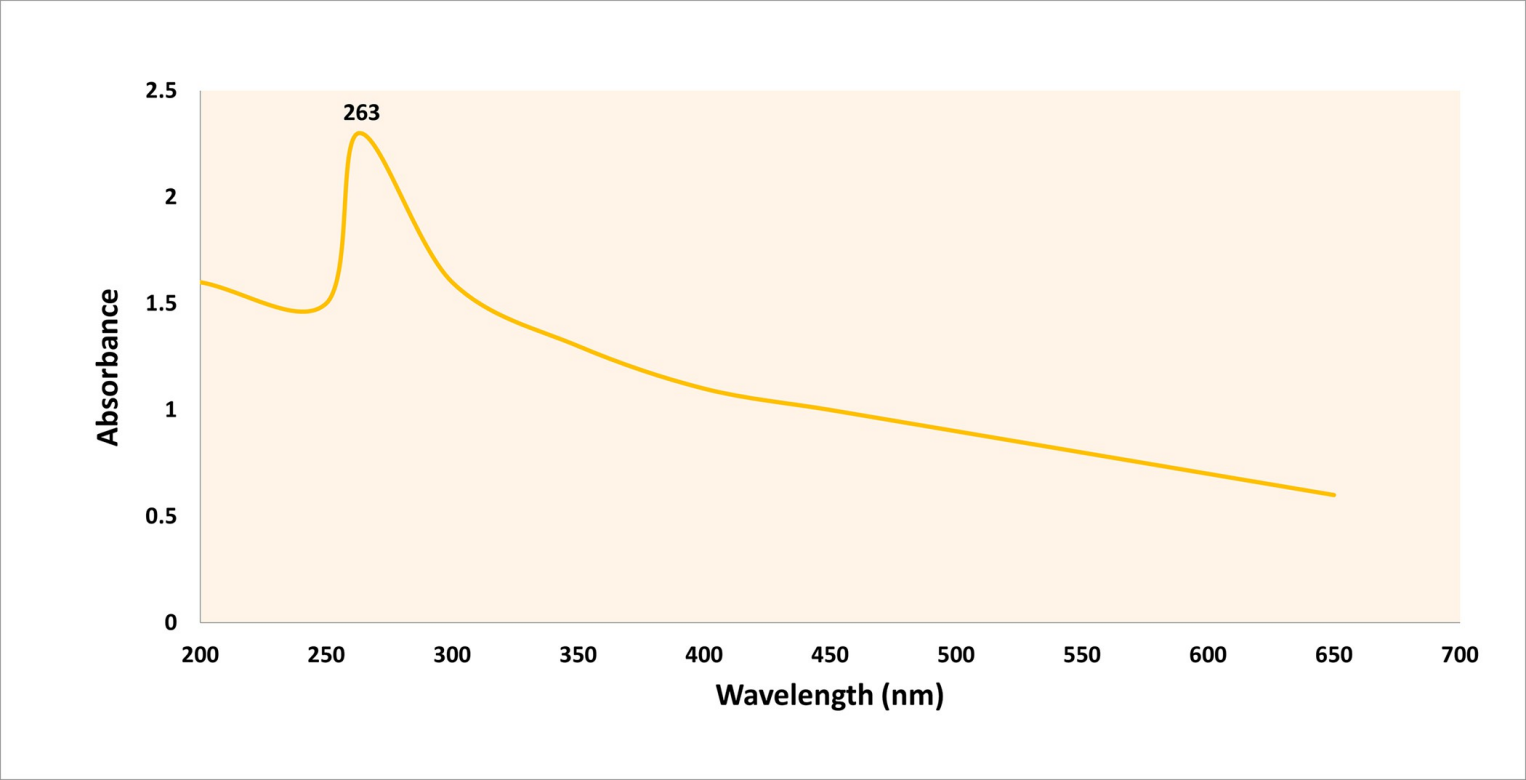
UV–visible spectrum of the green synthesized selenium nanoparticles (SeNPs).

The green synthesized SeNPs were characterized through UV-Visible spectroscopy, scanning electron microscopy (SEM), energy-dispersive X-ray (EDX), X-ray diffraction (XRD), and fourier transform infrared spectrometer (FTIR). Because a single technique is inadequate to analyze all the properties of the produced SeNPs, a combination of techniques is usually required. The most popular technique for studying the reduction and capping of SeNPs is UV-visible spectroscopy. In our study, a single absorption peak was observed at 263 nm through UV-Visible spectroscopy (Fig 1). According to Cittrarasu et al. [58], the Surface Plasmon Resonance (SPR) of SeNPs creates such a peak. The SPR is a resonance phenomenon induced by the interacting the conduction electrons of metal nanoparticles with incoming photons [59]. The single peak indicates the usual spherical morphology of nanoparticles [60]. Researchers have discovered that green synthesized SeNPs have UV-visible maximum absorption in various ranges in prior studies. Ramamurthy et al. [61] used fenugreek extract for SeNP synthesis and found an absorbance peak ranging from 200–400 nm, with the largest peak at 390 nm. In addition, SeNPs were synthesized from *Withania somnifera* of spherical morphology with a maximum absorbance of 310 nm by Alagesan and Venugopal [62]. Anu et al. [63]. produced SeNPs using garlic cloves and reported an absorption peak at 260 nm. Similarly, few researchers have described the synthesis of SeNPs using various reductant agents [64–66]. The first results of UV–visible spectroscopy revealed that *M*. *azedarach* leave extract was successfully reduced and stabilized the Na_2_SeO_3_ to SeNPs.

**Fig 2:**
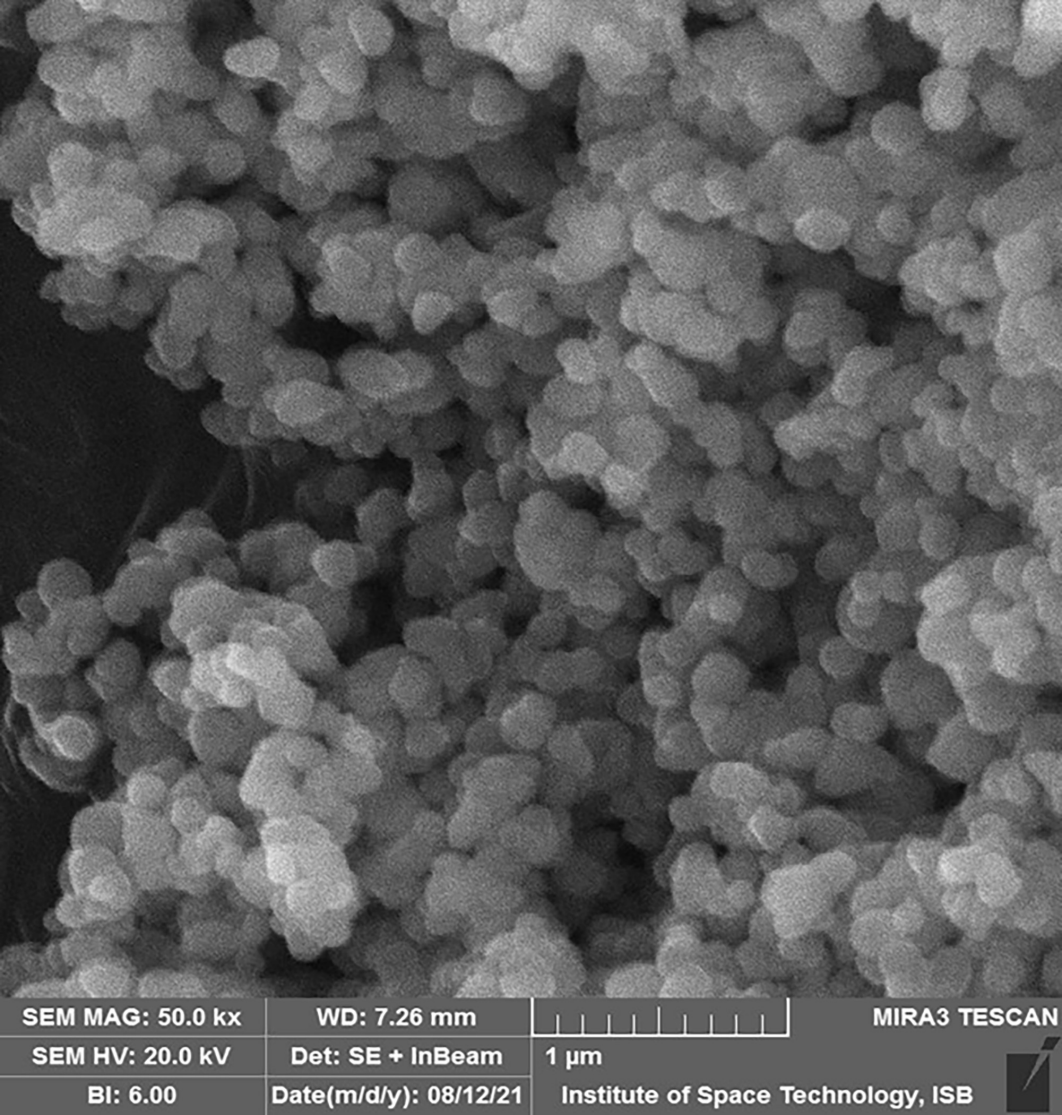
Scanning electron microscope (SEM) analysis of the green synthesized SeNPs.

The morphology of green synthesized SeNPs was confirmed by SEM which showed the spherical shape with an average size of approximately 74.43 nm (Fig 2). Our study agreed with Verma and Maheshwari [67], who reported a 74.25 nm average size of SeNPs. The results were like those of [57, 68, 69], they reported spherical SeNPs production from plant extracts. Researchers prepared SeNPs from leaf extracts of *Diospyros montana Capsicum annuum*, *Allium sativum*, and calculated SeNPs sizes of 80, 50–150, and 40–100 nm, respectively [61, 63, 70], which supports the current findings.

**Fig 3:**
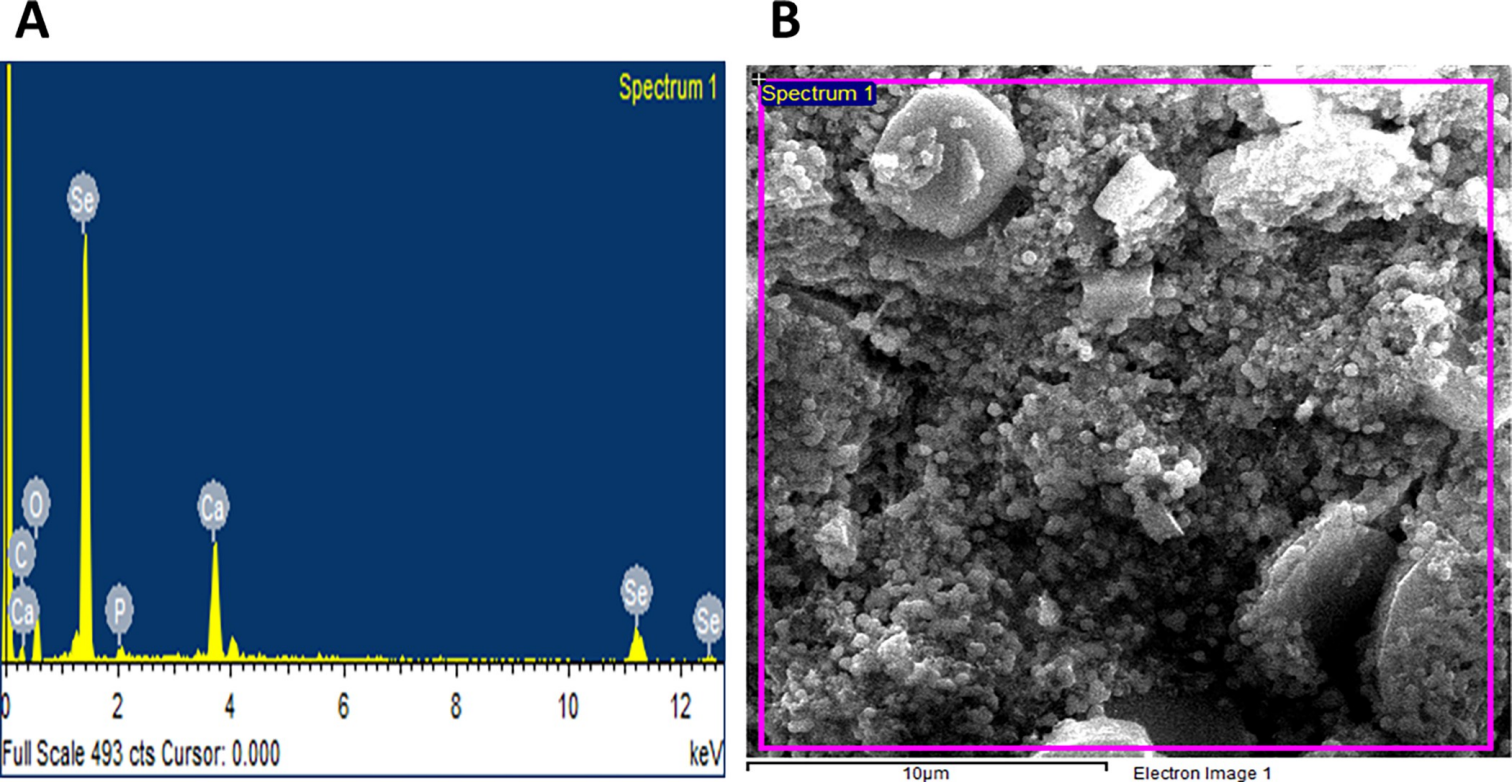
EDX spectrum of the green synthesized SeNPs.

EDX detector was used to confirm the presence of metallic selenium ions. The EDX spectrum elucidated strong absorption peaks of metallic selenium ions at 1.35 KeV, 11.20 KeV, and 12.40 KeV (Fig 3). SeNPs also contained reduced levels of O and C, which could be attributed to the flavonoids and phenolics found in the *M*. *azedarach* leave extract. Gunti et al. [57], who supported our research, used *E*. *officinalis* fruit extract for SeNPs synthesis and reported that SeNPs are made of Se, O, and C, with Se being the major element. Our findings were agreed with [71].

**Fig 4:**
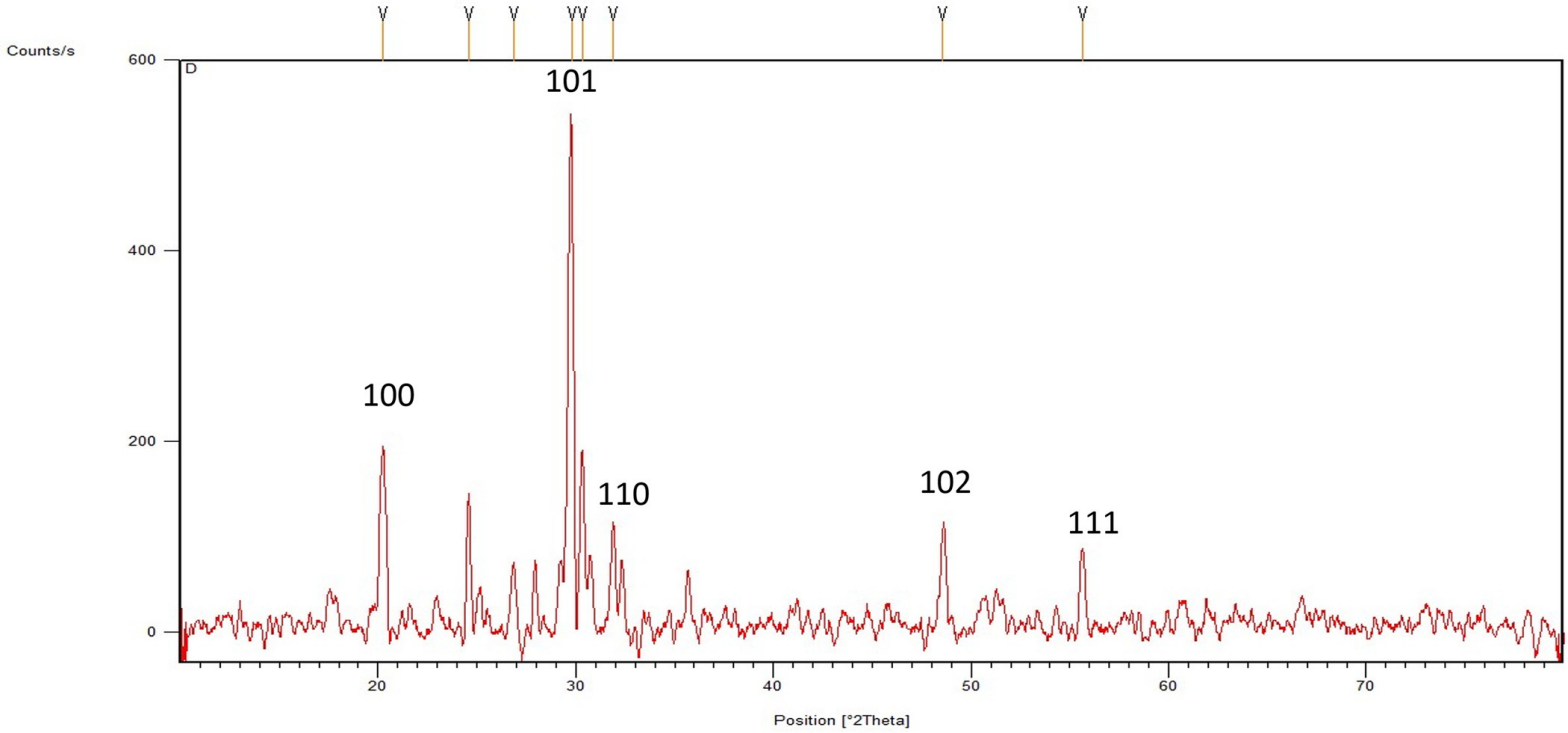
XRD diffractogram of the green synthesized SeNPs.

The crystalline nature of SeNPs was confirmed by XRD. The SeNP X-ray plans (100), (110), (101), (111), (102) were matched at diffraction peaks at 2Ɵ of 20.25°, 24.593°, 26.862°, 29.809, and 30.327° (Fig 4). These reflection plans reveal that the diffraction peaks were properly synchronized, and they were confirmed by the Joint Committee on Powder Diffraction Standards, file no. 06–0362 files, which clearly depict the crystalline nature of SeNPs. Previous studies [72] reported the crystalline SeNPs prepared from plant extract. Kazemi et al. [30] reported the crystalline nature of SeNPs prepared from starch solution. These findings are agreeing with obtained results from the study of Fresneda et al. [73] and Khan et al. [74] that reported the production of crystalline nanostructures. Our study is also in agreement with the works of Alam et al. [72], showing SeNPs obtained from *Withania somnifera* leaf aqueous extract were crystalline in nature.

**Fig 5:**
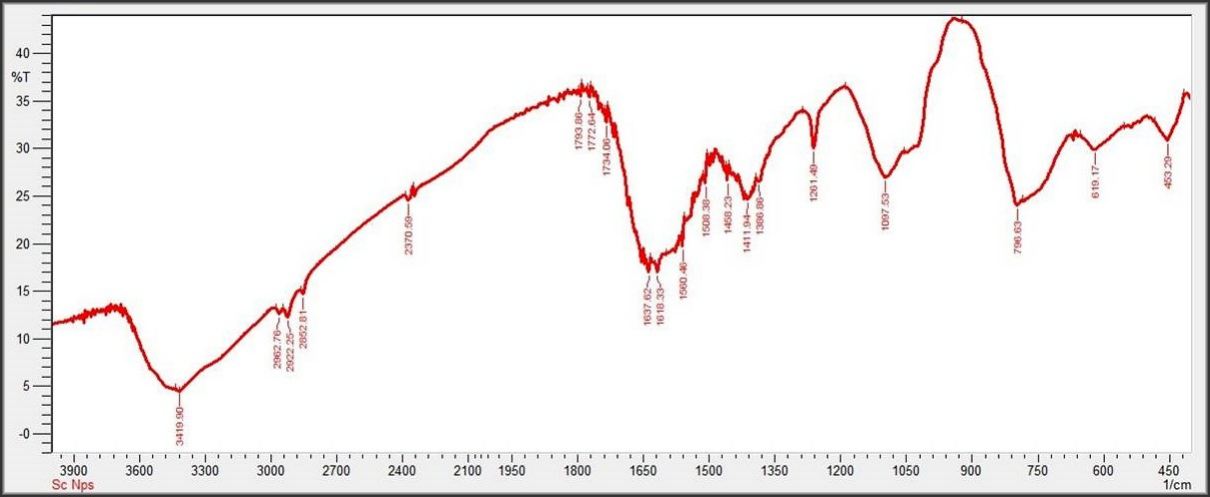
FTIR analysis of green synthesized SeNPs.

By measuring the chemical bond vibrational rates, the Fourier Transform Infrared spectrometer (FTIR) reveals the functional groups, present on the surface of the nanoparticles [74]. The complete spectra of *M*. *azedarach* leave extract and biosynthesized SeNPs are shown in Fig 5. The absorption peak at 3419.90 cm^–1^ corresponds to the hydroxyl group (O–H). The absorption peaks at 2962.76 cm^–1^, 2922.25 cm^–1^, and 2852.81 cm^–1^ represent the occurrence of carbon hydrogen stretching (C-H). Similarly, the peak at 2370.58 cm^–1^ confirmed the presence of carbon dioxide (0 = C = 0). The absorption peaks in 1793.86 cm^–1^, 1772.64 cm^–1^, and 1734.06 cm^–1^ represents the carboxyl groups. The absorption peaks at 1637.62 cm^–1^ and 1618.33 cm^–1^ relate to the C = C. The absorption peaks at 1560.46 cm^–1^ and 1508.38 cm^–1^ relates to the N-O and at 1458.23 cm^–1^ relates to the C-H. The absorption peaks at 1411.94 cm^–1^ and 1386.86 cm^–1^ relate to the S = O. The absorption peaks at 1261.49 cm^–1^ and 1097.53 cm^–1^ correspond to C-O. The absorption peaks at 796.63 cm^–1^, 619.17 cm^–1^, and 453.29 cm^–1^ confirmed the presence of C = C,C-Br, and C-Cl respectively. The different functional groups obtained through FTIR analysis of SeNPs synthesized by *M*. *azedarach* help in the reduction of biosynthesized SeNPs [75].

### 3.2. Effects of foliar applications of green synthesized SeNPs on physiological parameters of mango under the stress of mango malformation

**Fig 6:**
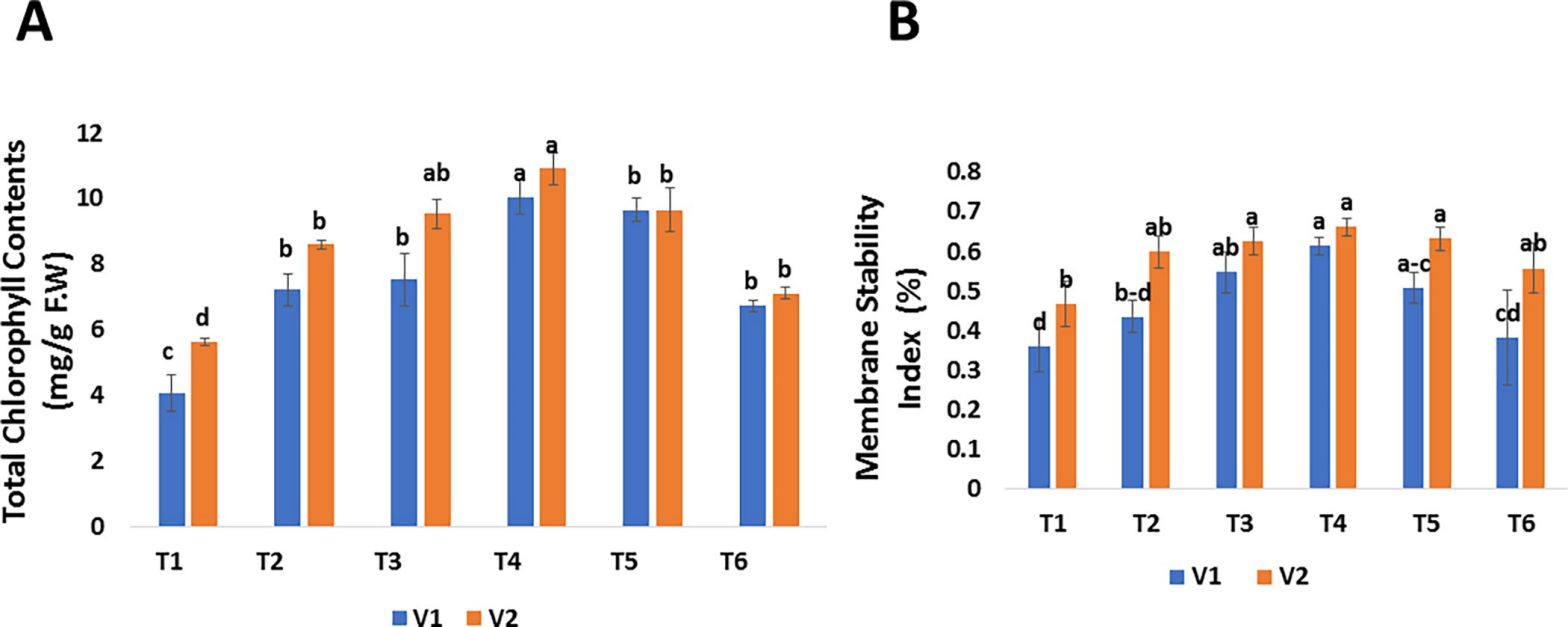
Effect of foliar applications of green synthesized SeNPs on physiological parameters of *Mangifera indica* under the stress of mango malformation disease. (A) Chlorophyll contents of *M*. *indica* in response to different treatments of SeNPs. (B) Membrane stability index of *M*. *indica* in response to different treatments of SeNPs.

The physiological parameters of mango plants were investigated to analyze the potential of green synthesized SeNPs against mango malformation-infected mango plants. Different concentrations of SeNPs (see Table 1) were applied as a foliar spray on the two varieties of mango (i.e., V1: Chaunsa and V2: Langra) and physiological parameters were recorded in terms of chlorophyll (photosynthetic pigment) and membrane stability index. The results of this study reported that the total chlorophyll content and membrane stability index of both varieties of mango were considerably increased. The highest chlorophyll contents were reported in both varieties (i.e., V1: 10.0587 mg/g F.W and V2: 10.9123 mg/g F.W) treated with 30 *μ*g/mL (T4) and the least quantity (i.e., V1: 4.0737 mg/g F.W and V2: 5.6247 mg/g F.W) was reported in untreated diseased mango plants (T1) where no SeNPs were applied (Fig 6A). The membrane stability index (%) was highest in both varieties of mango (i.e., V1: 61.43% and V2: 66.23%) treated with 30 *μg*/mL (T4) compared to untreated diseased mango plants (i.e., V1: 36.1% and V2: 46.8%) where no foliar application of SeNPs was sprayed (Fig 6B). The findings of this study are like those of Quiterio-Gutiérrez et al. [76]. Zahedi et al. [77], reported that SeNPs improved the chlorophyll content of tomato plants under *Alternaria solani* stress. Dong et al. [78], found that SeNPs increased the chlorophyll content in *Lycium chinense* leaves by 200–400%. Furthermore, SeNPs also increase the photosynthetic pigments in cluster beans, according to Ragavan et al. [79], Our results demonstrated that SeNPs enhanced the biosynthesis of chlorophyll contents. Selenium protects antenna complexes which in return increase the amount of photosynthetic content [80]. Ahmad et al. [81] also reported that Se protects mustard (*Brassica juncea* L.) plant under stress by means of increased starch accretion in the chloroplasts. Higher amounts of photosynthetic pigments were also detected in Se supplemented tomato plants [82]. The improvement in physiochemical activities of Se treated Chinese cabbage subsequently increased growth and development of plants [83] more tubers of larger size in case of potato crop [84, 85]. The present results agreed with those of Rady et al. [86], who reported that SeNPs promote physiological attributes against *Phaseolus vulgaris*. As a result, an increase in chlorophyll content in mango malformation-infected plants treated with SeNPs could help restore photosynthetic machinery and hence growth qualities. SeNPs also protect the chloroplast structure from severe oxidative damage, such as the breakdown of stroma and grana lamellae and speed up chlorophyll biosynthesis by protecting chloroplast enzymes [87]. Nasibi et al. [88] reported that pre-inoculation of fox tail seeds with SeNPs under stress, significantly increased chlorophyll content compared to plants grown under salinity stress without seed priming. Additionally, it has been communicated that Pseudomonas fluorescens strains ameliorated chlorophyll fluorescence and chlorophyll pigments in sweet corn under water-deficit stress [89] Also, ALKahtani et al. [90] observed that treatment of sweet pepper plants with PGPRs, increased the chlorophyll fluorescence and chlorophyll pigment.

### 3.3 Effects of foliar applications of green synthesized SeNPs on biochemical parameters of mango under the stress of mango malformation

**Fig 7:**
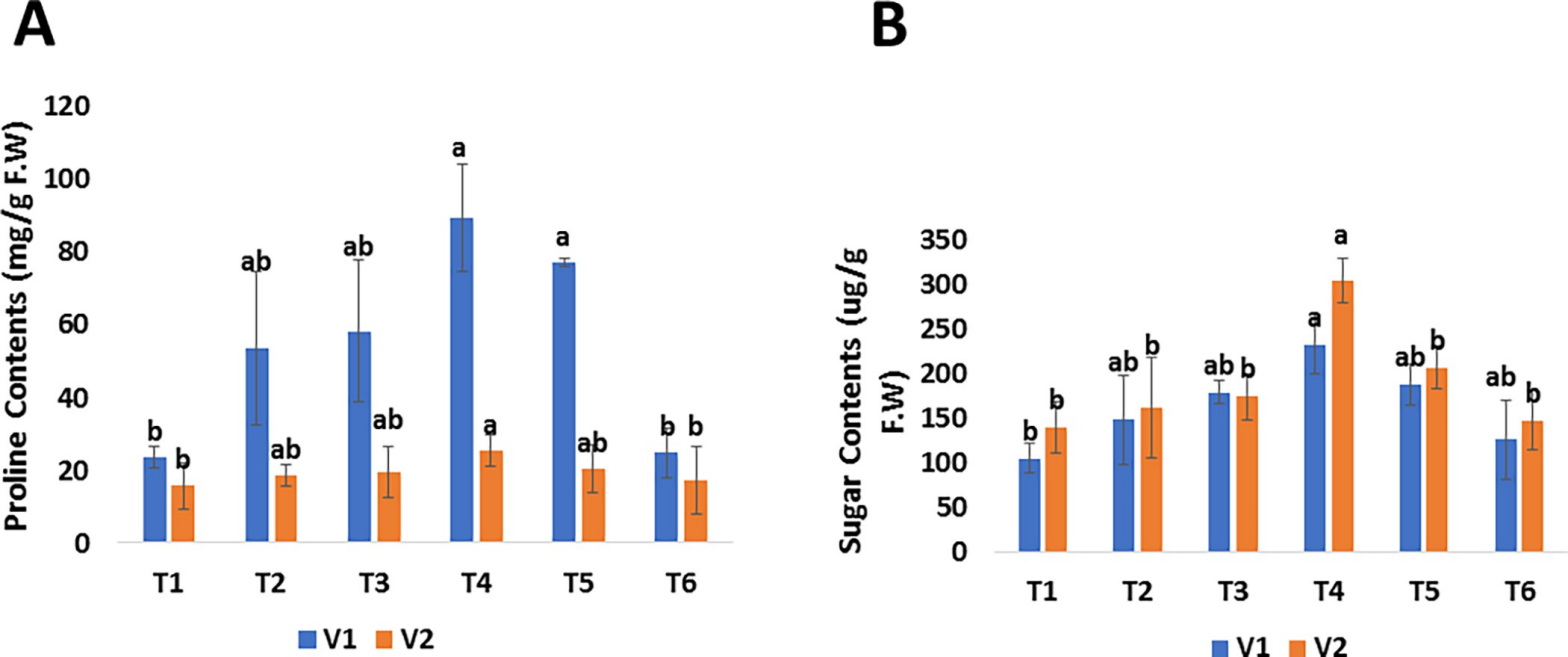
Effect of foliar applications of green synthesized SeNPs on biochemical parameters of *Mangifera indica* under the stress of mango malformation disease. (A) Proline contents of *M*. *indica* in response to different treatments of SeNPs. (B) Sugar contents of *M*. *indica* in response to different treatments of SeNPs.

The effects of foliar application of green synthesized SeNPs on biochemical parameters were recorded in terms of proline, and soluble sugar. The proline and soluble sugar contents were analyzed in untreated diseased plants and treated with different concentrations of SeNPs. The proline content was reported as the highest (i.e., V1: 89.257 mg/g F.W and V2: 25.369 mg/g F.W) in mango treated with 30 *μ*g/mL (T4) and the lowest proline content (i.e., V1: 23.407 mg/g F.W and V2: 15.651 mg/g F.W) was reported in untreated diseased mango plants (T1) where no SeNPs were applied (Fig 7A). Furthermore, our findings show that mango malformation disease has a significant impact on plant-soluble sugar concentration. Under the stress of mango malformation, low soluble sugar contents (i.e., V1: 104.91 *μ*g/g and V2: 139.83 *μ*g/g) were analyzed in both varieties of mango. The amount of soluble sugar was increased due to the foliar spray of SeNPs. The highest soluble sugar was reported in both varieties of mango (i.e., V1: 233.65 and V2: 85.64 *μ*g/g F.W) treated with 30 *μ*g/mL (T4) spray of SeNPs (Fig 7B). Proline helps in the protection of plants from oxidative damage and the maintenance of an osmotic environment. Proline also protects proteins against denaturation when they are exposed to harsh circumstances [91]. Similarly, soluble sugars play an important part in the defense systems of plants. Sugars are the fundamental substrate for plant defense responses, supply structural material and energy. Sugar content also affects the plant immune system by acting as a signal molecules that interact with hormone signaling. Green synthesized SeNPs improved the proline and sugar content in mango plants under the stress of malformation, according to the current data from the field experiment. The findings are consistent with those of Zahedi et al. [77], who found that 20 mgL^–1^ of SeNPs increased osmolyte synthesis in strawberry plants under stress circumstances. Likewise, Sardar et al. [17], reported that plants raised from seed primed with SeNPs enhanced the proline and soluble sugar contents. The current study supports the findings of prior research [36, 86, 92]. Nasibi et al. [88], reported that the amount of proline and soluble sugar in foxtail millet plants increased under salinity stress. Proline levels in plant tissues have likely increased because of higher production, less usage in protein synthesis, and enhanced protein hydrolysis as well as decreased proline degradation. The amount of proline under control and the salinity stress were both raised by pretreating foxtail millet seeds with bacteria and a solution of selenium nanoparticles. In addition, SeNPs treatment boosted proline content in both control and Cd-stressed plants, according to Sardar et al. [17], It appears that the SeNPs increased the proline synthesis by increasing the activity of nitrate reductase, which is necessary for proline synthesis [93] According to Shahid et al. [94], selenium increases the production of soluble sugars by improving the cytoplasmic membrane’s integrity and decreasing malondialdehyde, which promotes overall growth.

### 3.4 Effects of foliar applications of green synthesized SeNPs on antioxidant defense system of mango under the stress of mango malformation

#### 3.4.1 SOD and POD

**Fig 8:**
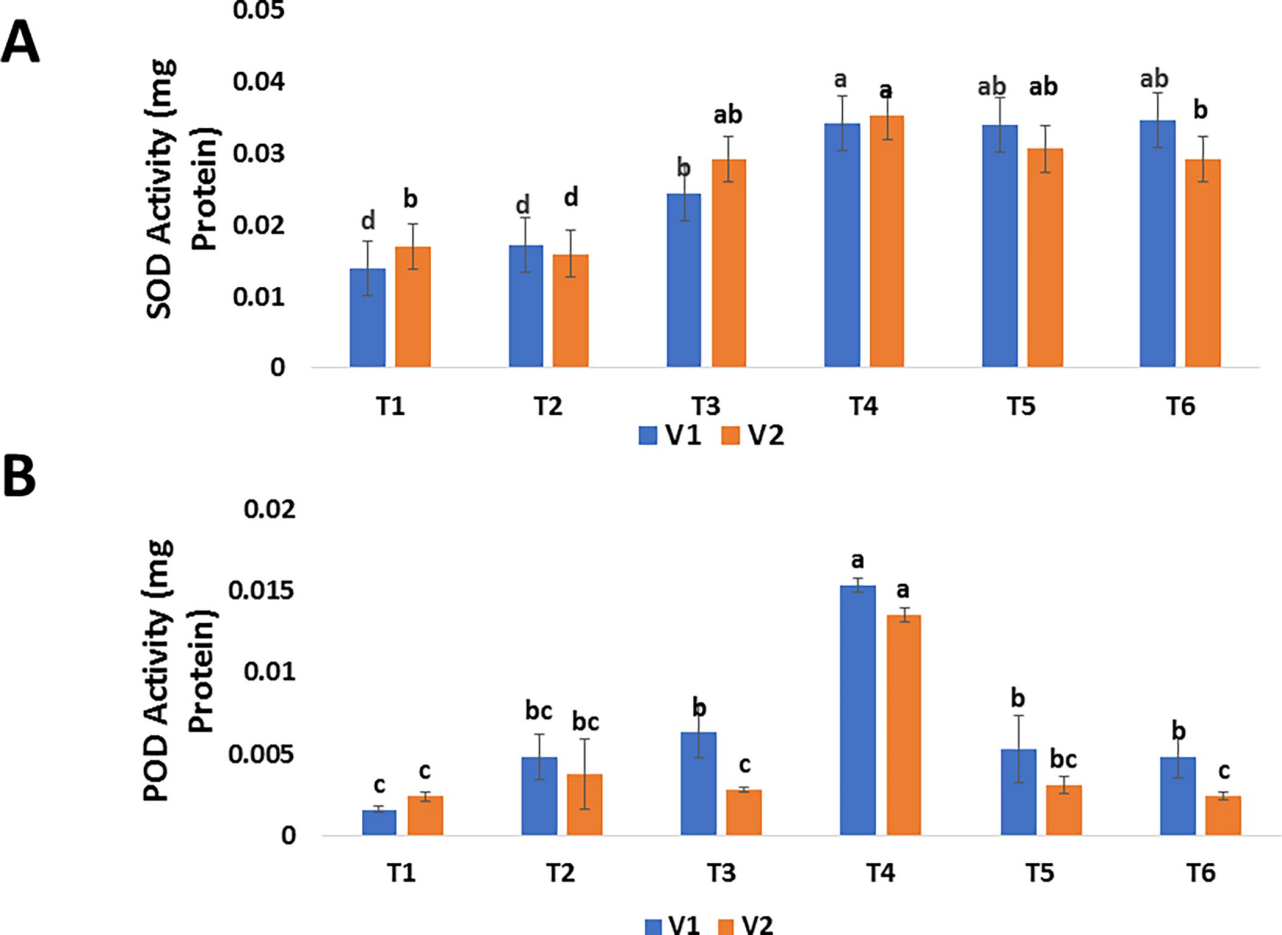
Effect of foliar applications of green synthesized SeNPs on biochemical parameters of *Mangifera indica* under the stress of mango malformation disease. (A) SOD activity of *M*. *indica* in response to different treatments of SeNPs. (B) POD activity of *M*. *indica* in response to different treatments of SeNPs.

The activities of enzymatic antioxidants such as superoxide dismutase (SOD), and peroxidase (POD) were considerably increased by green synthesized SeNPs. SOD and POD activities of untreated diseased plants were recorded as 0.014 mg protein (VI), 0.017 mg protein (V2), and 0.0016 (V1), 0.0024 mg protein (V2) respectively. The findings revealed that green synthesized SeNPs had distinct effects on mango malformation afflicted plants by modifying antioxidant and enzyme activities to counteract the negative effects. The results showed that the 30 *μ*g/mL increased the level of SOD, and POD as 0.0343 mg protein (V1), 0.0353 mg protein (V2) and 0.0153 (V1), 0.0135mg protein (V2) respectively (Fig 8A & 8B). Plants and animals rely on their immune systems to protect them from diseases that can be deadly. Immune-related disorders are common in animals, but they are infrequently investigated in plants. Mango malformation disease drastically reduced the levels of enzyme activities such as SOD and POD, according to the findings of present study. Exogenous foliar applications of green synthesized SeNPs increased the levels of defense-related activities in mango malformation-affected mango plants compared with untreated mango plants, according to the findings. The findings of this work are consistent with those of previous studies. SeNPs increased the level of SOD in stressed sorghum plants, resulting in greater stress tolerance [95]. Similarly, several additional investigations have found that SeNPs increased POD and SOD activities in stressed strawberry plants [77]. Jiang et al. [96], found that selenium treatments triggered antioxidant defense genes and enhanced the content of SOD in corn, resulting in higher stress tolerance in plants. Gunti et al. [57] evaluated the antioxidant activity for phyto-fabricated SeNPs, and found it has excellent antioxidant activity. Our results regarding SOD and POD activity of enzymes are near about findings of Hussein et al. [97].

#### 3.4.2 Phenolic and flavonoids

**Fig 9:**
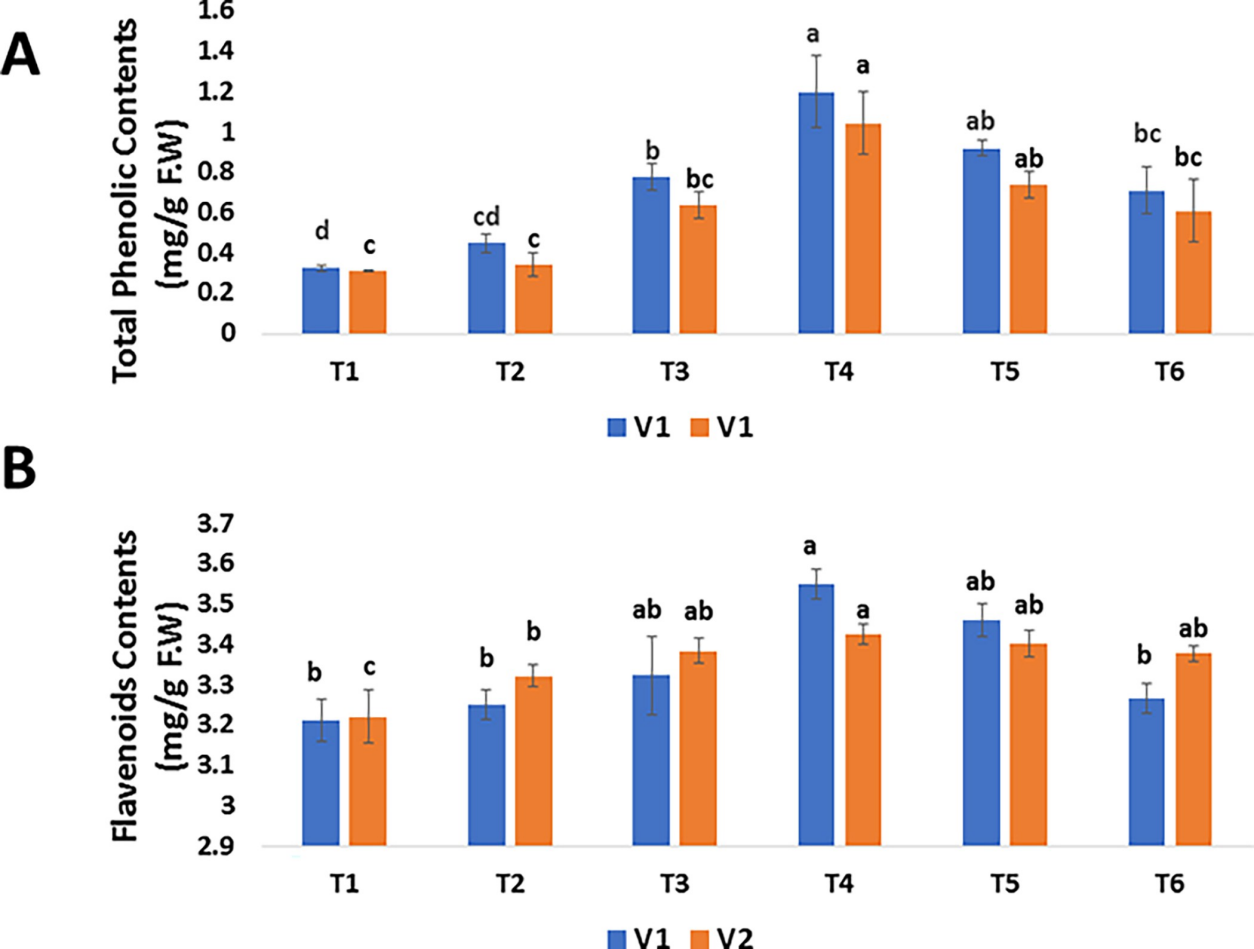
Effect of foliar applications of green synthesized SeNPs on biochemical parameters of *Mangifera indica* under the stress of mango malformation disease. (A) Total phenolic contents of *M*. *indica* in response to different treatments of SeNPs. (B) Total flavonoid contents of *M*. *indica* in response to different treatments of SeNPs.

Phenolic (i.e., V1:1.2003 *μ*g/mg DW and V2: 1.0437 *μ*g/mg DW) and flavonoids (i.e., V1: 3.5493 *μ*g/mg DW and V2: 3.4233 *μ*g/mg DW) contents were comparatively higher in both varieties treated with 30 *μ*g/mL SeNPs than in untreated diseased plants (i.e., V1: 0.3257 *μ*g/mg DW and V2: 0.03133 *μ*g/mg DW) and (i.e., V1: 3.2107 and V2: 3.2203 *μ*g/mg DW) respectively (Fig 9A & 9B). According to the findings, SeNPs at 30 *μ*g/mL showed outstanding outcomes and were shown to be the optimal concentration of SeNPs for improving the antioxidant defense system of mango malformation diseased mango plants by up—regulating enzymatic and non-enzymatic activities.

Plants are subjected to various biotic and abiotic stressors, which alter the electron transport system, which is participating in the production of reactive oxygen species (powerful oxidizing agents that destroy plant cells) [98]. Plants produce enzymatic and non-enzymatic substances that neutralize ROS and defend cells from oxidative damage to protect themselves from the harmful effects of ROS [99]. Plants’ natural response to stress is the synthesis of non-enzymatic antioxidants such as phenolic and flavonoids compounds [100]. Many studies have found that using various nanoparticles can creates antioxidant chemicals, which can help plants resist pathogens. Mango malformation illness reduced the levels of non-enzymatic chemicals in mango malformation afflicted plants, according to our findings. This is because malformation diseases have the most lethal impact on plants’ antioxidant defense mechanism. However, our findings demonstrated that applying SeNPs to mango malformation-affected plants increased the synthesis of flavonoids and phenolic compounds compared with untreated mango malformation infected plants. The results of the present study agreed with the previous studies [86, 92, 97]. The current findings are consistent with those of Quiterio-Gutiérrez et al., [76]. Similarly, the current findings support the findings of Lopez-Vargas et al. [101], who found that CuNPs raised flavonoids in tomato of about 36.14 percent. Shahraki et al. [102] reported that application of nano-Se significantly increased the antioxidant activity of leaves and flowers under non-saline (30 and 4%) and saline (12 and 22%) conditions compared to the control, respectively. Guleria et al. [103] reported the strong antioxidant activity of SeNPs. Previous studies by Kondaparrthi et al. [104] Mellinas et al. [105] Boroumand et al. [106] Dumore et al., [107] reported the strong antioxidant activity of green synthesized SeNPs.

### 3.5 Isolation of *F*. *mangifera* and assessment of *in vitro* antifungal activity and inhibition percentage of green synthesized SeNPs

**Fig 10:**
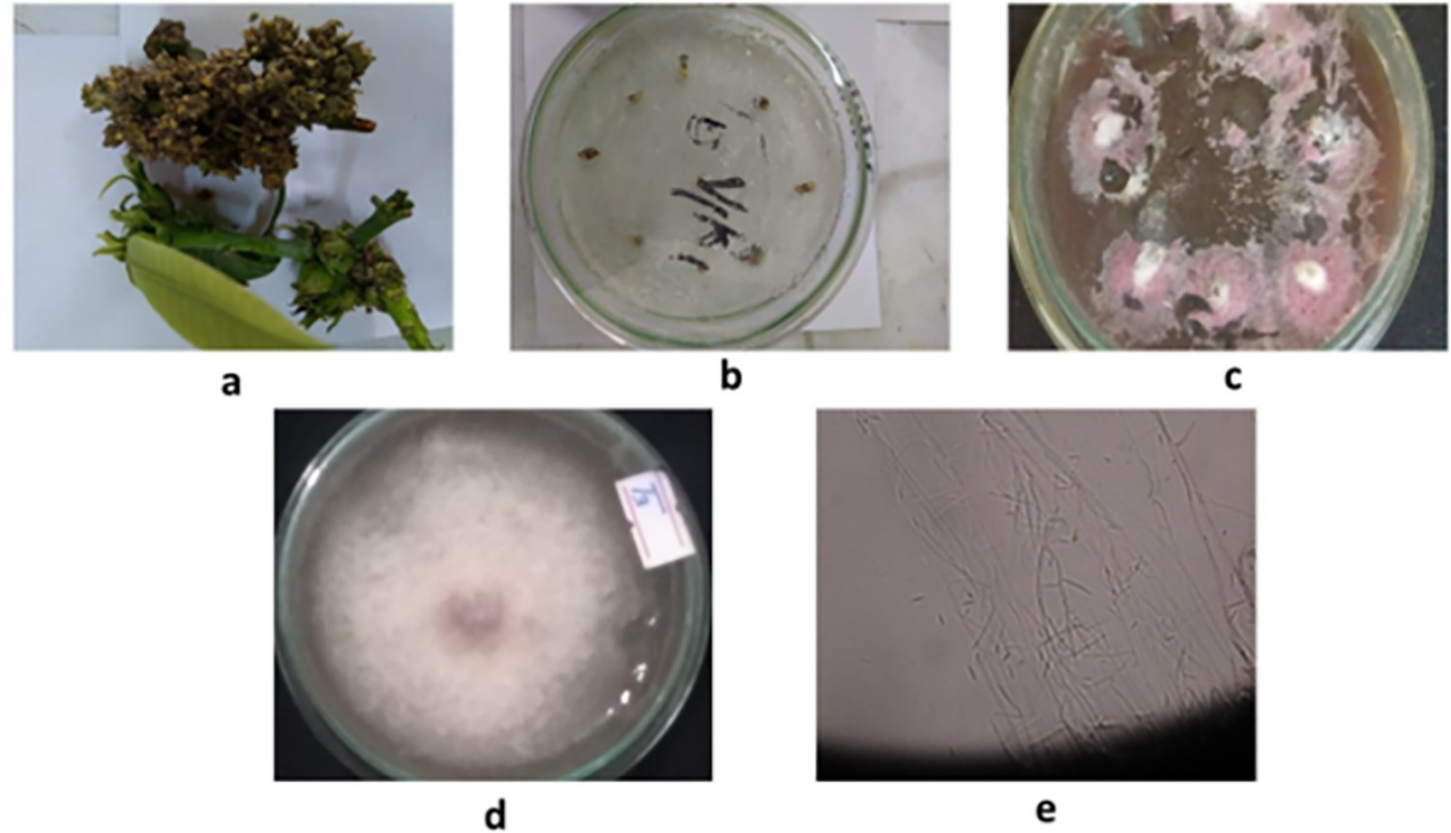
Procedure for isolation and identification of *Fusarium mangifera* causing mango malformation disease. (a) Infected inflorescence of *Mangifera indica* (b) Infected inflorescence were placed on PDA, (c) fungal colonies emerging from the inflorescence after 3–5 days at 28 ± 2°C under alternative cycle of darkness and light in a Versatile Environmental Test Chamber, (d, e) Macro and micro morphological examination.

The isolated fungus showed slow to moderate growth on PDA. Initially, the fungus exhibited the white to cream colored colonies producing aerial mycelium and cotton like resemblance. Micro-conidia were found to be short to medium in length, oval to ellipsoidal in shape, aseptate, hyaline, and borne on polyphialides, with a size range of 7.8 X 2.8m. Macroconidia have a thin wall, are falcate, have 2–3 septa, and are 25 X 42 m in size (Fig 10). Our findings are consistent with earlier research [108–110].

#### 3.5.1 Assessment of *in vitro* antifungal activity and inhibition percentage of green synthesized SeNPs against *Fusarium Mangifera*

**Fig 11:**
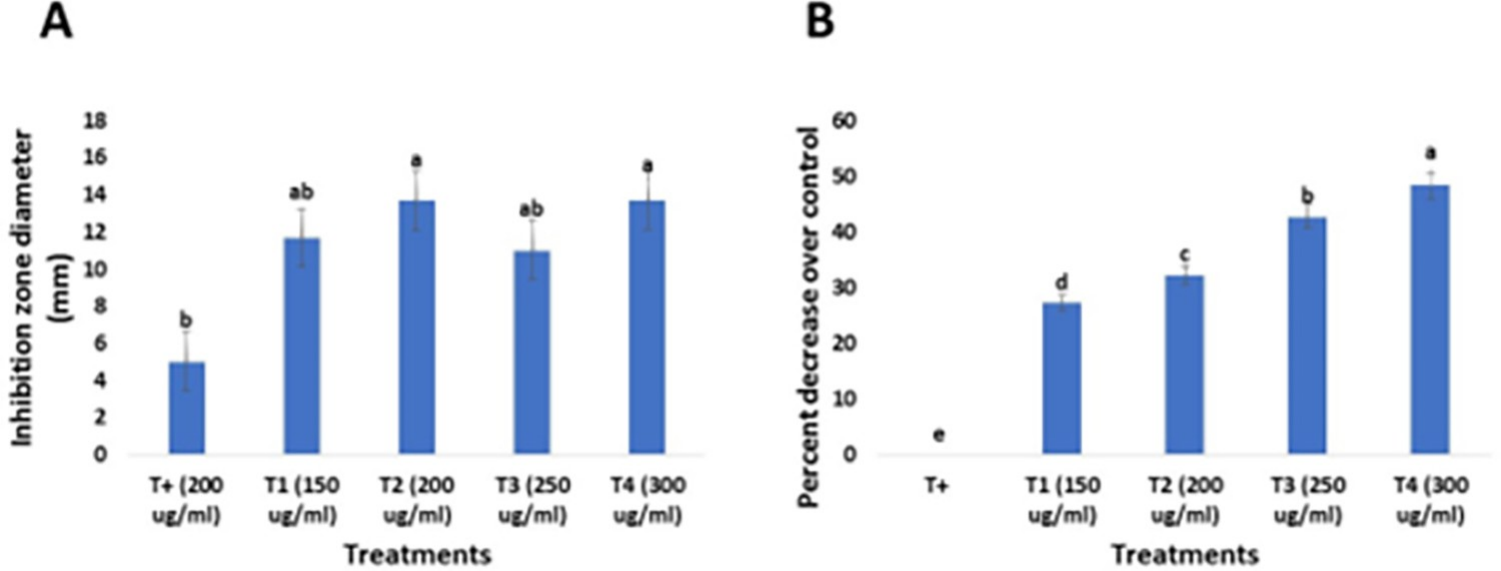
Antifungal activity of green synthesized SeNPs against *Fusarium mangifera*. (a) Well diffusion method (b) Radial growth method. Data are expressed as means ± standard deviations of triplicate assays. The different alphabetic superscripts in the same column are significantly different (p < 0.05) based on Duncan’s multiple comparison test.

The antifungal activity of SeNPs was evaluated against *F*. *mangifera* using the agar well diffusion method by using different concentrations i.e., 150, 200, 250, 300 *μ*g/mL and the results were compared with the antifungal drug Barresten (standard). The results illustrated that different concentrations of SeNPs had antifungal activity against *F*. *mangifera*. It was reported that as we increased SeNPs concentrations, the inhibition zone diameter was also increased (Fig 11A). SeNPs at 300 *μ*g/mL (T3) showed the maximum antifungal activity with an inhibition zone of 14 mm, whereas the antifungal drug Barresten showed the lowest antifungal activity against *F*. *mangifera* with an inhibition zone of 5 mm. The inhibition percentage for each concentration of SeNPs against *F*. *mangifera* was reported by the radial growth method. The percentage of inhibition increased as the concentration of SeNPs increased, according to the results. The inhibition percentage was 48.31 percent at a concentration of 300 *μ*g/mL, and this concentration had the least fungicidal activity. Furthermore, SeNPs at 250 *μ*g/mL had a substantial inhibitory percentage but not as high as 300 *μ*g/mL (Fig 11B).

**Fig 12:**
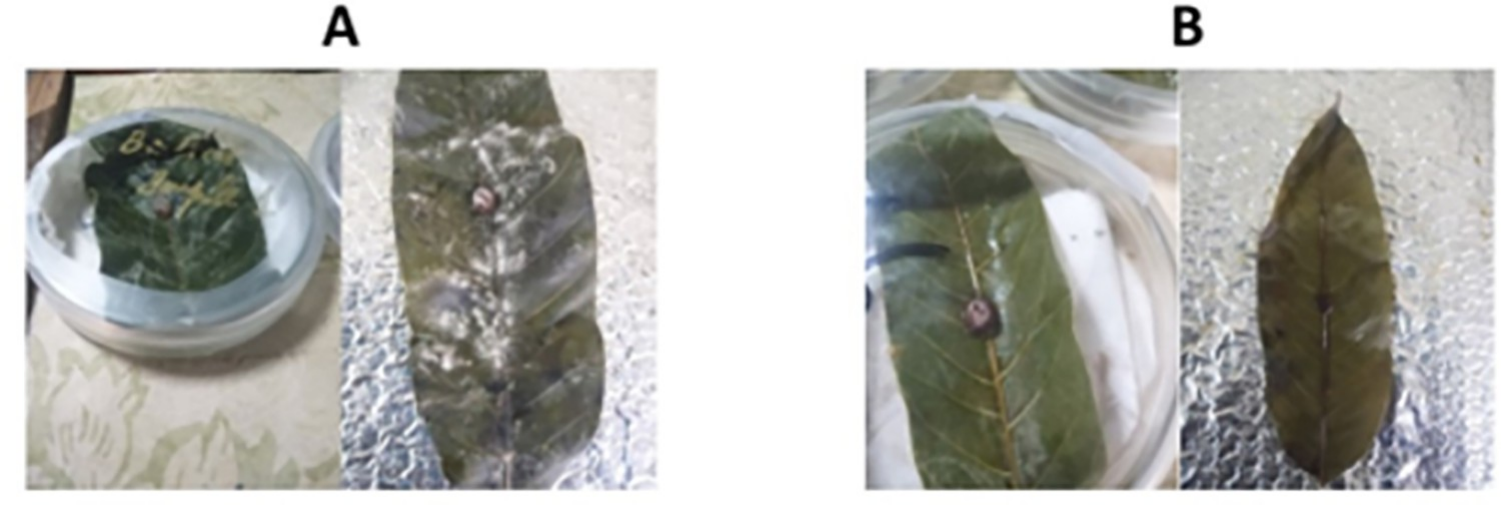
*In vitro* leaflet assay of green synthesized SeNPs against *Fusarium mangifera*.

**Fig 13:**
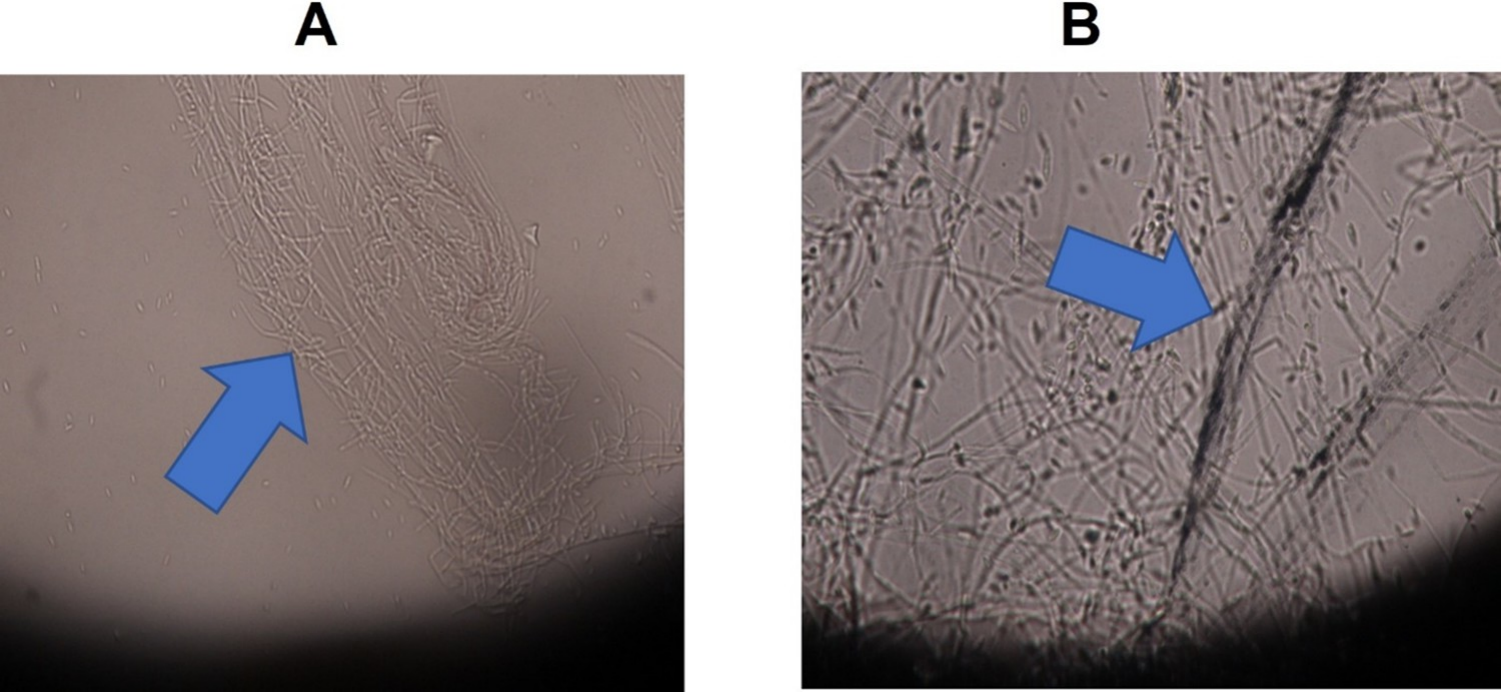
Cavity slide assay of green synthesized SeNPs against *Fusarium mangifera*.

The best treatment 300 *μ*g/mL was selected for further antifungal analysis by the *In vitro* leaflet assay and cavity slide method. In the *in vitro* leaflet assay, one month old, dried mango leaf was primed with 300 *μ*g/mL concentrations of SeNPs and then artificially inoculated with *F*. *mangiferae*. Control was maintained without priming with SeNPs. It was reported that the pathogen was spread on the leaf surfaces without the application of SeNPs (Fig 12A). In other hands the leaf that was primed with 300 *μ*g/mL concentration of SeNPs proved to be efficient in controlling the spread of the fungus on the leaf surface (Fig 12B). In the cavity slide method, the inhibitory effect of SeNPs on *F*. *mangifera* can be clearly observed in Fig 13A where conidial germination was inhibited when the fungal suspension was treated with 300 *μ*g/mL SeNPs compared to the control where 100% conidial germination was measured. Overall inhibitory effect of SeNPs on *F*. *mangiferae* was checked by making a slide of control and treated with SeNPs and observed under a light microscope. The healthy and clearly disintegrated mycelium was seen in Fig 13B.

AgNPs [111, 112], CuNPs [113], and ZnNPs [114] are commonly used to control fungal plant diseases. SeNPs on the other hand, have potent antifungal properties but are rarely used to combat fungal plant infections. *Sclerospora graminicola* growth and proliferation could be inhibited by biosynthesized Se-NPs [115]. One of the aims behind the antifungal effects of SeNPs may invade the cell membrane and disrupt cell membrane integrity. Thus, the damaged cell membrane could lead to the leakage of vital cell materials and causing cell death [116–119]. Kazemi et al. [30]. reported the antifungal activity of SeO_2_-NPs to prevent the growth of *T*. *tonsurans*, *T benhamiae*, *T*. *rubrum*, *T*. *interdigitale*, *M*. *canis*, *T*. *mentagrophytes*, *M*. *fulvum*, and *E*. *floccosum*. Furthermore, SeNPs were used to suppress *Alternaria alternate*-caused tomato leaf blight, with SeNPs at a dose of 100 *μ*g/mL resulting in an inhibition percentage of 89.6% [120]. SeNPs applied against *Alternaria solani* induced Early Blight Disease in potatoes, with a 100% inhibition rate at 80ppm [121]. The results of the present study agreed with that of Joshi et al., [53]. Our results are supported by previous studies. Abdel-Moneim [52], used SeNPs against different phytopathogenic fungi. Vrandecic et al. [122], reported the strong antifungal activity of SeNPs stabilized with different coating agents Ali et al. [123], reported the antifungal activity of SeNPs against *Candida albicans*. In studies with *Candida glabrata* it has been found that SeNPs attach and agglomerate on the surface of cells, cause loss of membrane’s smoothness, appearance of bulges and impairment of membrane’s integrity [124]. Vasylchenko and Derevianko [125] reported the strong antifungal activity of Selenium and iodine nanoparticles against Fusarium spp. Strong antifungal activity of green synthesized SeNPs was reported by Hashem et al., [126] Moreover, Lashin et al. [127] confirmed SeNPs that biosynthesized from Ziziphus spina-christi exhibited antifungal activity against unicellular and multicellular fungi. Salem et al. [128] reported that the biosynthesized SeNPs from pomegranate peel extract have promising antifungal activity. Safaei et al. [129] reported that green synthesized showed the best performance and prevented more than 70% of fungal growth. Previous research revealed that selenium had antibacterial action at the nanoscale. Due to their special qualities, including a high surface to volume ratio, specific surface area, quantum effects, increased surface reactivity, and unique chemical and physical characteristics, metal NPs have been developed as antimicrobial agents. By reacting with the thiol (-SH) protein group, which affects membrane permeability, DNA damage, oxidative stress, mitochondrial membrane malfunction, changed gene expression, and altered cell shape, nanoparticles can kill resistant infections [130, 131]

## 4. Conclusions

The results of present study explained the effect of SeNPs on plant physiology and biochemistry. Mango trees under stress of vegetative malformation responded positively in both physiologically and biochemically with 30 *μ*g/mL foliar application of SeNPs. As a result, plant physiological and biochemical attributes were improved. Under *in vitro* study, SeNPs also played a significant role to inhibit the *F*. *mangiferae* growth. The maximum fungal inhibition was noted at concentrations of 300 *μ*g/mL of SeNPs. Hence, it is proved that SeNPs can play a vital role in both in vivo and *in vitro* condition to improve the plant physiological and biochemical attributes. Therefore, SeNPs possesses excellent antifungal activity. There is a need to increase usage of nanoparticles to control the diseases of mango especially mango malformation. Biogenic selenium NPs are widely expected to be efficient and cost-effective treatments for fungal plant diseases. Before its commercial usage in plant disease control in the field, the adverse effects of these biogenic NPs on agriculture and ecosystems should be determined.
